# Geographical information system and access to HIV testing, treatment and prevention of mother-to-child transmission in conflict affected Northern Uganda

**DOI:** 10.1186/1752-1505-1-12

**Published:** 2007-12-03

**Authors:** Dick D Chamla, Olushayo Olu, Jennifer Wanyana, Nasan Natseri, Eddie Mukooyo, Sam Okware, Abdikamal Alisalad, Melville George

**Affiliations:** 1 World Health Organization, formerly with Health Leadership services (HLS), 20 avenue appia, Geneva 1211, Switzerland; 2 Uganda country office, World Health Organization, 24578 Kampala, Uganda; 3 Health Resource Center, Uganda Ministry of Health, Kampala, Uganda; 4 Community Health department, Uganda Ministry of Health, Kampala, Uganda; 5 Regional Office for Africa, World Health Organization, Brazzaville, Congo

## Abstract

**Objectives:**

Using Geographical Information System (GIS) as a tool to determine access to and gaps in providing HIV counselling and testing (VCT), treatment (ART) and mother-to-child transmission (PMTCT) services in conflict affected northern Uganda.

**Methods:**

Cross-sectional data on availability and utilization, and geo-coordinates of health facilities providing VCT, PMTCT, and ART were collected in order to determine access. ArcView software produced maps showing locations of facilities and Internally Displaced Population(IDP) camps.

**Findings:**

There were 167 health facilities located inside and outside 132 IDP camps with VCT, PMTCT and ART services provided in 32 (19.2%), 15 (9%) and 10 (6%) facilities respectively. There was uneven availability and utilization of services and resources among districts, camps and health facilities. Inadequate staff and stock-out of essential commodities were found in lower health facility levels. Provision of VCT was 100% of the HSSP II target at health centres IV and hospitals but 28% at HC III. For PMTCT and ART, only 42.9% and 20% of the respective targets were reached at the health centres IV.

**Conclusion:**

Access to VCT, PMTCT and ART services was geographically limited due to inadequacy and heterogeneous dispersion of these services among districts and camps. GIS mapping can be effective in identifying service delivery gaps and presenting complex data into simplistic results hence can be recommended in need assessments in conflict settings.

## Background

Delivery of HIV counseling and testing (VCT) and antiretroviral services for prevention of mother to child transmission (PMTCT) and for long-term treatment (ART) to eligible individuals is feasible in emergency settings [1]. Northern Uganda, which has been affected by 20 years of the Lord's Resistance Army (LRA) insurgency, has experienced internal displacement of persons (IDP) into camps, disruption of health services and high HIV prevalence [2-4]. In Gulu, Kitgum and Pader (Acholi sub-region), which are the most conflict-affected districts in Northern Uganda, HIV/AIDS was the second most frequently reported cause of death in 2005 [5]. While VCT has been ongoing for the past decades, PMTCT and ART services have been scaled up more recently after the declaration of the World Health Organization (WHO) strategies of 3 by 5 and Universal Access. However, little has been documented on the access to these services in this conflict affected region.

In health, defining access precisely has been difficult [6]. However, various models, such as Penchansky's typology of access have recently increased a general understanding of this concept [6]. In line with these models, this article describes access to VCT, PMTCT and ART in the context of their availability and utilization. We then link this information on availability to the Geographical Information System (GIS). GIS is defined as computer based systems for integration and analysis of geographical data through mapping [7]. Though recent increase in its use for health assessments and policies has been evident [7], yet, limited information is available on its application in conflict settings. This led the authors of this article to use GIS to map the availability of VCT, PMTCT and ART services located inside and outside the IDP camps in Northern Uganda with the aim of determining access to these services for guiding priority setting, scaling up strategies and resource allocation.

## Methods

**Figure 1:**
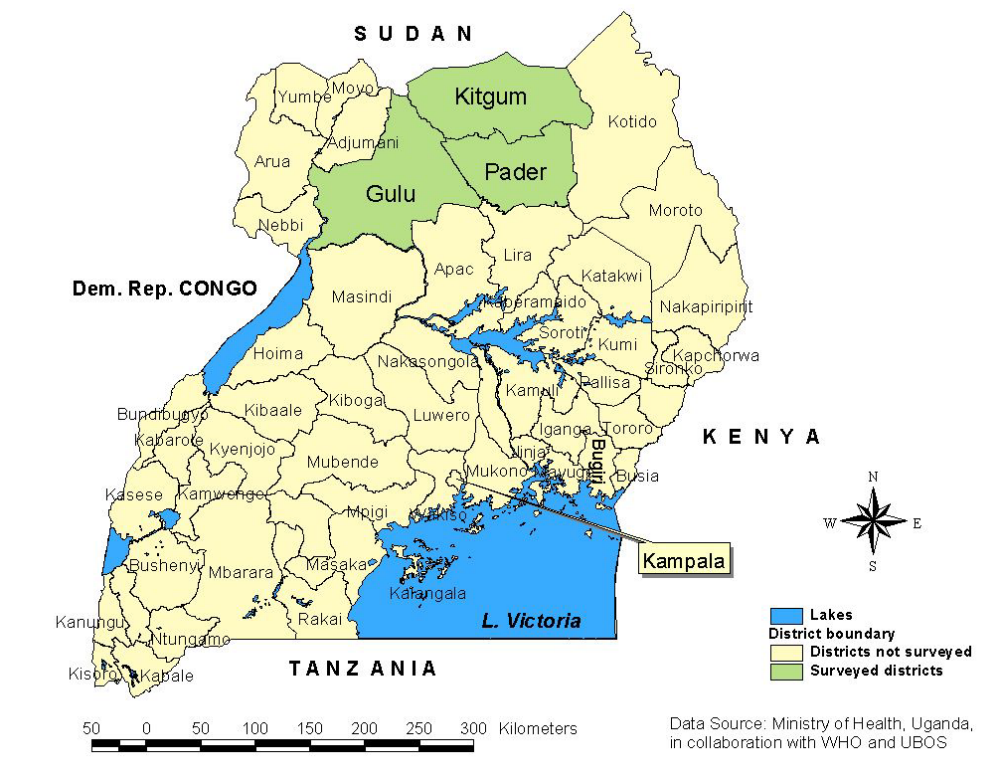
Uganda map showing the surveyed Northern districts of Gulu, Kitgum and Pader.

We performed Service Availability Mapping (SAM) of health facilities located inside and outside the IDP camps in three districts (figure 1): Gulu (camps = 65, population = 543,267); Kitgum (camps = 43, population = 322,781) and Pader (camps = 24, population = 373,035). District population figures were projected using 2002 census report at an annual growth rate of 3.4% [8]. The SAM consisted of a survey methodology whereby structured camp and health facility questionnaires were used to collect data in the field, and the Global Positioning System (GPS) instruments which were used to collect the geo-coordinates of the camps and health facilities as in WHO SAM guidelines [9]. These GPS readings were then uploaded to and processed by the GIS software to generate the maps.

Data collection extended from 8^th ^to 30^th ^April 2006 and all camps and health facilities located inside and outside camps in the three surveyed districts were mapped. Teams of two trained local interviewers administered standardized, pre-piloted camp and health facility questionnaires respectively (in English language). The main respondent for the camp questionnaire was the camp commandant or his assistant. For the health facility, the main respondents were the health facility in-charges and heads of VCT, PMTCT and ART if these services were available in that health facility. The camp questionnaire collected camp geographical coordinates (longitudes, latitudes and altitudes), administrative location and division, population figures, availability of health facilities, and human resources for health. The health facility questionnaire collected the facility geographical coordinates, availability of VCT, PMTCT and ART services as well as utilization of these services based on the review of the records of the patients' attendance in the previous one month (30 days) as in Pappa E *et al *[10]. The geo-coordinates were taken at the administrative point designated for meetings in the camp as identified by the camp commandant whereas in the case of health facility the coordinates were taken 10 meters away from the main administrative building of that health facility.

As in Roberts *et al *[11], we measured access in two ways: first by assessing physical availability of VCT, PMTCT and ART services and their minimum essential components based on the national Antiretroviral guidelines [12] and compared the availability of these services to population, camp and health facility level. The minimum essential components for VCT service were presence of at least 1 trained health worker, HIV test kit and a register. For PMTCT, the minimum components for this service included the presence of a trained staff, HIV test kits, Nevirapine and intra- and post-partum care. For ART services, presence of a medical officer, HIV test kits and anti-retroviral drugs (ARVs) formed the minimum essential components. Secondly, the access was measured by the utilization of VCT, PMTCT and ART services based on the assessment of clients' attendance records during the past 30 days as explained above. These records were available from health facility registers that are part of Uganda Health Management Information System (HMIS). We did not assess demographic characteristics of those clients attending these services. Similarly, as there were no user fees for most services including VCT, PMTCT and ART [13,14], we concluded that affordability had little effect on utilization. Likewise, in northern Uganda, most IDP camps are of small geographical size characterized by over-crowded IDP dwellings which are in close proximity to most health services [5]. This means all VCT, PMTCT and ART services inside the camps or municipality were located within accessible distances. However, as the level of violence was observed more outside the camps [5,14], we assumed that insecurity rather than distance had likely effect on utilization of those services located outside or in another camp. Since all three districts were in the same security phase as classified by United Nations (phase III), we presumed that there was no significant differences in the level of violence that would effect utilization differently among the districts.

In order to estimate and compare the size of the population receiving these services among districts, we divided the monthly utilization figures per estimated number of eligible individuals in a district per 1000 persons. For PMTCT, the number of eligible pregnant women was estimated by multiplying district antenatal HIV prevalence with a projected pregnant population (which is 5.2% of the district population based on projected census figures). During the survey, the antenatal HIV prevalence for Gulu, Kitgum and Pader was 11.9%, 7.2% and 11% respectively [13]. Since no district specific HIV sero-prevalence data was available, we estimated ART eligible individuals by multiplying the number of people infected with HIV/AIDS using northern Ugandan region seroprevalence rate of 8.2% [4] by 15% which is the percentage of HIV infected individuals who are eligible for ART [13]. For VCT, we compared the utilizations by estimating the proportions of clients' attendance and the district population aged 15–49 years of age (39.1% of population based on census projections) per 1000 persons. Since this study did not include the conflict-free control region from another part of Uganda for comparison, instead we compare our findings with the targets set by Uganda Health Sector Strategic plan (HSSP II). This also determined the gaps in service provision.

Data from the three districts were entered on Visual FoxPro 7 (Microsoft Corp, Redmond, WA, USA) and analyzed using SAS 8.1 (SAS Institute, Cary, NC, USA). After merging the three district datasets, data was exported to Microsoft Excel, then saved as Dbase before being exported to ArcView GIS 3.3 (ESRI) software to produce maps for the geographical locations of camps and those health facilities offering VCT, PMTCT and ART services. In this study, the health facility was considered *functional *when there was a facility structure, supplies and service providers with evidence of service utilization as shown in the client registers.

### Ethical approval

The study received an approval from the Uganda Ministry of Health and the offices of the district directors of health services in all the three districts. The methodology was also peer reviewed and approved by Uganda Ministry of Health and Bureau of Statistics.

## Results

In total, there were 167 health facilities and 132 IDP camps (population ranging from 200 to 54,610) in the three surveyed districts. Of the total health facilities, 119 (71.3%) were functional [health center II (HC II) = 65; health center III (HC III) = 39; health center IV (HC IV) = 7; hospitals = 8] with Gulu having 60 (50.4%), Kitgum 25 (21%) and Pader 34 (28.6%) facilities.

### Voluntary Counseling and Testing (VCT)

**Figure 2:**
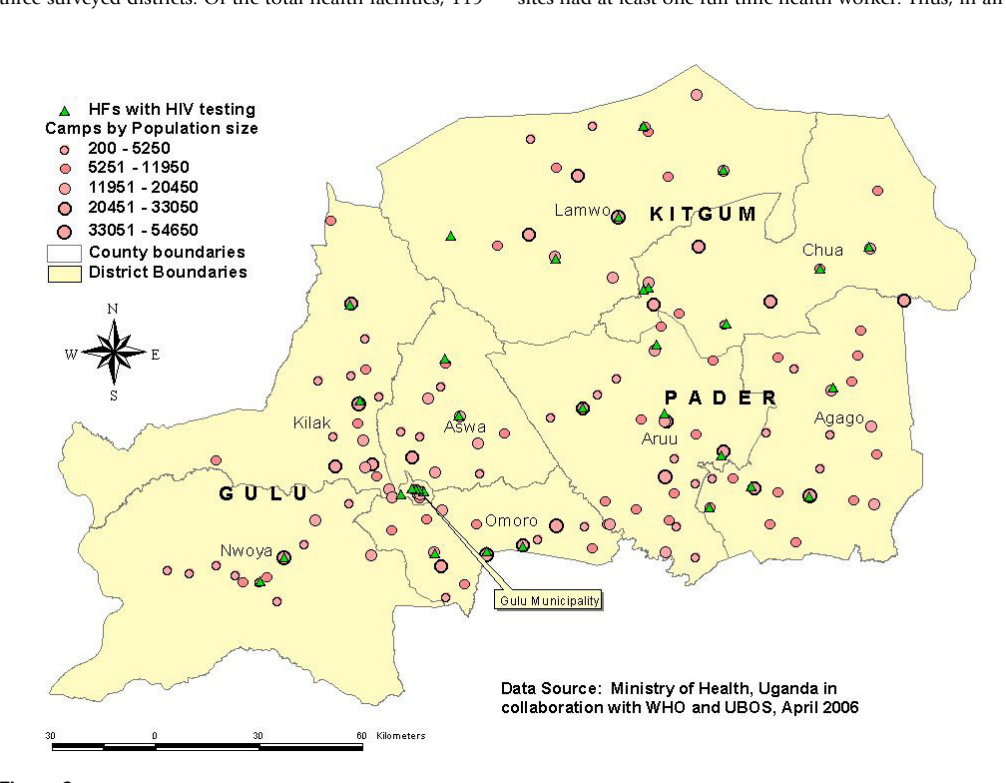
Availability and distribution of VCT services according to the IDP camps in Gulu, Kitgum and Pader districts, April 2006.

Figure 2 shows the availability and distribution of VCT services by camps. As shown, VCT sites were evenly distributed across the counties except in the southern parts of Kilak, Aswa and Aruu counties. Of the 14 VCT sites in Gulu district, 5 (35.7%) were located in the municipality. Availability of VCT sites according to health facility level is presented in table 1. Of the 32 total VCT centers in Acholi sub-region, 11 (34.3%) were located in health center III and the rest were evenly distributed among other health facility levels.

**Table 1:** Availability of VCT, PMTCT and ART according to health facility level in Acholiland

**Health facilities**	**VCT**	**PMTCT**	**ART**
Health center II	6 (18.8%)	1 (6.7%)	0 (0%)
Health center III	11 (34.3%)	4 (26.7%)	1 (10%)
Health center IV	7 (21.9%)	3 (20%)	1 (10%)
Hospitals	8 (25%)	7 (46.6%)	8 (80%)
Total	**32**	**15**	**10**

Similarly, 7 (21.9%) of the total VCT sites reported stock-out of HIV testing kits on the day of the survey. All VCT sites had at least one full time health worker. Thus, in all the three districts, 2 (33.3%) of VCT sites in HC II had all minimum components; the percentages were 8 (72.7) for HC III, 6 (85.7%) for HC IV and 8 (100%) for hospitals.

Data on utilization showed that during the previous one month prior to the survey, there were 9314 clients who received HIV counseling in all the three districts, however out of total clients counseled, 6772 (72.7%) were tested for HIV. In Gulu and Kitgum, the proportions of clients per districts' populations aged 15–49 years who had HIV testing in the last month were 18 and 17 per 1000 persons respectively, while in Pader this proportion was 6 per 1000 persons.

### Prevention of Mother to Child Transmission of HIV (PMTCT)

**Figure 3:**
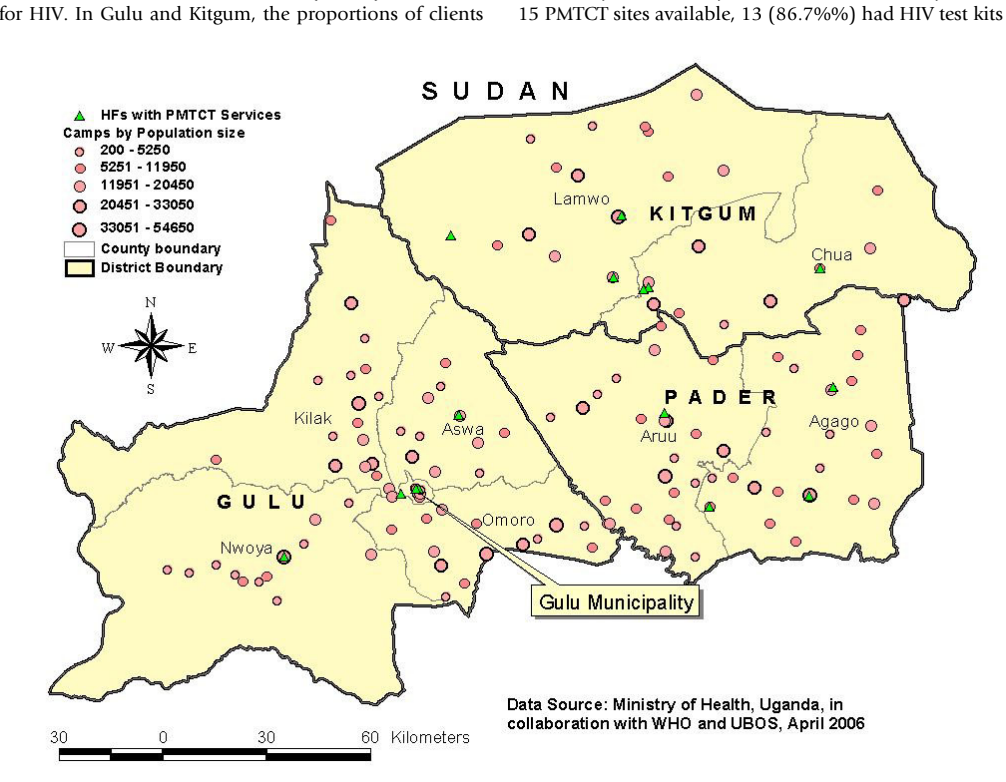
Availability and distribution of PMTCT services according to the IDP camps in Gulu, Kitgum and Pader districts, April 2006.

The availability and distribution of PMTCT sites according to the camps is presented in figure 3. As shown, PMTCT sites were equally distributed among districts but unevenly distributed among counties and camps. In Gulu, of the 5 PMTCT sites available, 3 (60%) were located in the municipality while other counties such as Kilak and Omoro with a total of 30 camps (populations ranging from 200 to 54,650), did not have PMTCT site. Similarly, Northern side of Kitgum district closer to Sudan border with 9 camps (populations ranging from 200 to 33050) did not have any PMTCT site. In table 1, which shows the availability of PMTCT by the level of health facility, of the 15 PMTCT sites available, 13 (86.7%%) had HIV test kits on the day of the survey and 14 (93.3%) had intra- and post-partum facilities and none of the PMTCT sites reported stock-out of Nevirapine. All sites had at least one service provider for PMTCT services. Similarly, of the total PMTCT sites, 13 (86.7%) had all minimum essential components. Based on clients' records in the previous one month [Gulu = 212; Kitgum = 50 and Pader = 9], there were an estimated 63, 41 and 4 per 1000 eligible pregnant women in Gulu, Kitgum and Pader respectively who received Nevirapine for PMTCT.

### Antiretroviral Treatment (ART)

**Figure 4:**
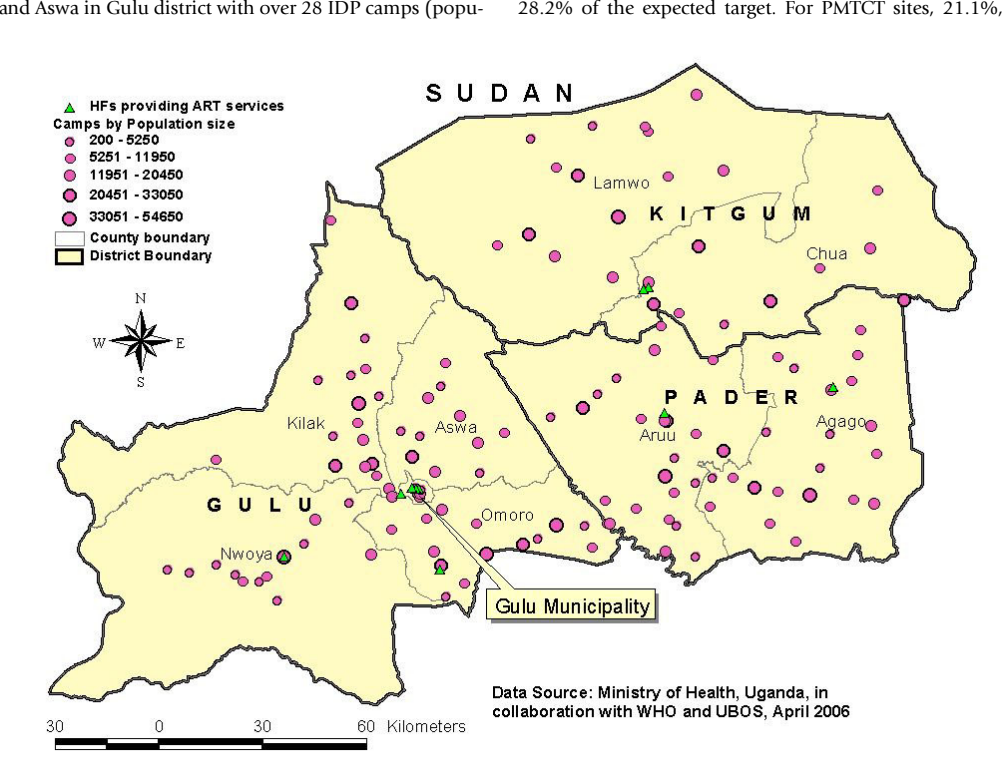
Availability and distribution of ART services according to the IDP camps in Gulu, Kitgum and Pader districts, April 2006.

Figure 4 shows the availability and distribution of ART sites according to the camps in the Acholi sub-region. The figure shows that, ART sites were unevenly distributed among districts and counties except in Pader district where the 2 available ART sites were distributed in two counties. Of the 6 ART sites in Gulu district, 4 (66.7%) were located in the municipality. Also, 2 counties of Kilak and Aswa in Gulu district with over 28 IDP camps (populations ranging from 200 to 54,650) did not have ART site. Similarly in Kitgum district, all 2 ART sites available were located in the municipality. Based on table 1, of the total 10 ART sites, 8 (80%) had at least one medical officer, 9 (90%) had HIV test kits, 6 (60%) had ARV available and 4 (40%) reported stock-out of ARV on the day of survey. Only 6 (75%) of ART sites in the hospitals had all minimum essential components while neither ART sites in HC III nor IV had all the components. Based on clients' records in the previous one month [Gulu = 146; Kitgum = 472 and Pader = 10], there were an estimated 22, 119 and 2 per 1000 ART eligible individuals in Gulu, Kitgum and Pader respectively who received ART.

### The gaps in service provision

The gaps in VCT, PMTCT and ART service provision are summarized in tables 2. Based on HSSP II targets, there was 100% availability of VCT services at HC IV and hospitals, however at HC III, VCT services availability was 28.2% of the expected target. For PMTCT sites, 21.1%, 42.9% and 87.5% of HSSP II targets were reached at HC III, HC IV and hospitals respectively. Similarly, 20% and 100% of HSSP II targets for provision of ART services were reached at HC IV and hospitals respectively.

**Table 2:** Availability of VCT, PMTCT and ART services compared to HSSP target

Health facility level	Functional health facilities	Number of VCT sites	HSSP target for VCT*	Number of PMTCT sites	HSSP target for PMTCT**	Number of ART sites	HSSP target for ART***
HC II	65	6		1			
HC III	39	11 (28.2%)	39	4 (21.1%)	19	1	
HC IV	7	7 (100%)	7	3 (42.9%)	7	1 (20%)	5
Hospitals	8	8 (100%)	8	7 (87.5%)	8	8 (100%)	8
Total	119	32 (59.3%)	54	15 (44.1%)	34	10 (66.7%)	15

* HSSP II target is 100% of HC III and above to have VCT services

** HSSP II target is 50% of all HC III and 100% at HC IV and above to have PMTCT services

*** HSSP II target is 75% of all HC IV and 100% of hospitals to have ART services

## Discussion

This was the first comprehensive mapping and assessment that determined access to VCT, PMTCT and ART services in a conflict affected Northern Uganda. Given the large population affected by this conflict coupled with high HIV/AIDS prevalence, the results of this study confirms the limited access due to inadequacy and uneven distribution of these services among the districts, counties and camps. Complicating the situation is insecurity, ongoing establishments of new settlements and large number of IDP camps scattered in a vast geographical area in these districts. Among the three districts, access to VCT, PMTCT and ART services was relatively better in Gulu evidenced by both, large number of facilities providing these services and utilizations. The likely reasons might be its comparatively large population size and longer humanitarian crisis that attracted most of relief assistance including provision of HIV related services. Similarly, Gulu as a former headquarters for Acholi sub-region, has a well-developed infrastructure system with relatively more capacity to support humanitarian operations.

The finding that most VCT, PMTCT and ART services were clustered in urban areas is consistent to the literature [13,14]. This geographical inequity has left most camps and rural areas lacking these services. Conversely, while VCT services were evenly provided across health facility levels, PMTCT and ART were mostly available at Health center IV and hospitals. This might be due to the fact that VCT has been ongoing in Uganda for decades while PMTCT and ART have been recently introduced. Another reason is the policy issue as in Uganda ART services are required to be provided at HC IV and above, with a medical officer being a pre-requisite [6,10] while for PMTCT, the policy requires this service to start at HC III and above [10].

Availability of minimum essential components also shows a similar pattern of inadequacy at lower health facility levels. The results of the stock-outs of HIV test kits and drugs at lower health facilities reflect limited capacity of supply chain system probably as a result of insecurity or funding. Similarly, shortage of health staff for ART might be due not only to inadequate number but mal-distribution of this cadre within and among the districts. For instance in Gulu, 79% of all medical officers work in the hospitals within the Municipality and 86% of those in Pader work in one hospital [15]. As most of these hospitals are located in municipalities, this further indicates urban-rural disparities in the access particularly to PMTCT and ART in northern Uganda.

Based on our data, there is evidence of utilization of VCT, PMTCT and ART services in all the districts. For VCT, the data showed a significant proportion of those counseled received HIV testing, however, our study did not assess the characteristics or reasons for those clients who did not take HIV testing. Our results also show that most PMTCT utilization was observed in Gulu despite an even distribution of PMTCT sites among districts. This is likely due to the presence of large number of hospitals compared to Kitgum and Pader reflecting the possible differences in quality of services. The paradoxical finding of ART utilization in which Kitgum had highest proportion of individuals on ART in the last month despite the limited number of ART sites should be interpreted cautiously. Without data on monthly trends in utilizations, this finding can be misleading. Yet, for Pader district the consistent lowest utilizations of VCT, PMTCT and ART shown by this study is likely driven by the inadequacy of services and resources including stock-outs of medicines as reported by other studies in the past [5,13-15]. However, other factors such as demographic and insecurity differences which have not been elucidated by this study, might also be the likely explanation.

In health system perspective, access defined in terms of physical availability and service utilization, is one of the intermediate outcome measures which has been increasingly used to determine health system performance as it influences both, health status and client satisfaction [9]. In this context, our findings of limited access to VCT, PMTCT, ART might be a reflection of limited HIV/ART system performance in this region. The findings of other studies on high prevalence and mortality due to HIV/AIDS in Northern Uganda [4,5], might likely be proximately related by limited access of these services as depicted by this study. Similarly, the gaps observed by this study, underpins the importance of scaling up of VCT, PMTCT and ART in this sub-region. The main challenge however is that most of the health facilities in rural areas or camps comprise of lower health facility levels which lack appropriate health personnel and medicines to offer services such as PMTCT and ART. Critical to this is therefore a review of current policies including the recruitment and retention of appropriate staff so that services like PMTCT and ART can be rolled out to lower-level health facilities.

Despite the evidence that application of GIS methodology in emergency settings is limited [16], still there were no studies which determined the feasibility of its use in these settings. In our study, the training on the use of GPS receivers, which were ordered locally, was done by Uganda authorities indicating that the local expertise is available. The analysis of data and production of maps was also accomplished locally. Training and complete data collection in this insecure and wide geographical area took less than 1 month. These indicated that using GIS as a tool in health assessments in conflict settings is feasible and can be locally undertaken.

Our finding of access can be limited by several factors. The use of last month facility attendance for measuring and comparing utilization among the districts can be event driven and might not accurately represents monthly average or variations in utilizations. This type of data can not give users' perspective on utilization and may lack comprehensive information of other determinants of utilization such as demographic variations, acceptability or user-satisfaction. Shortage of staff at lower health facilities might likely impair data collection hence underreporting utilizations. Using district population to estimate and compare utilizations among districts is also likely to be misleading. The use of catchment populations which the facility sub-served would have increased the reliability of our comparisons. Moreover, absence of the comparison district which is conflict-free, is another limitation as it would have determined whether the limited access to and gaps in providing VCT, PMTCT and ART services in northern Uganda was attributed to the longstanding conflicts. It is therefore essential to further examine other factors that affect access particularly the correlates of utilization of these services in this conflict region so as to achieve the goal of universal access by 2010.

In conclusion, the study shows that access to VCT, PMTCT and ART services in northern Uganda is geographically limited due to inadequacy and uneven availability and utilization of these services among districts, health facilities and camps with Pader district mostly affected. Addressing the gaps depicted by this study requires policy review, equitable geographical re-distribution or recruitment of appropriate staff and scaling up plans focusing on essential minimum components of services at lower health facilities. This study has shown that measuring access in emergencies not only provides information for health policy and planning but supplements information related to health system performance and health status of the population. Similarly, application of GIS for health need assessments in conflict settings is feasible and maps can be effective in presenting large set of data into simplistic, visual friendly and easily interpretable information.

## Competing interests

The author(s) declare that they have no competing interests.

## Authors' contributions

Dick Chamla designed the study protocol, ensured quality of data, assisted in data analysis and wrote the first draft of this article. Olushayo Olu contributed to the protocol development, mobilized resources, supervised data collection and assist in writing this article. Jennifer Wanyana supervised field implementation of this study, data collection, data analysis and contributed to the writing of the article. Natseri Nasan trained interviewers in the use of GPS, produced maps and contributed to the methodology section of the article. Melville George and Abdikamal Alisalad provided overall central supervision of the study, organized financial resources from donors, reviewed the protocol and contributed to the article writing. Eddie Mukooyo and Sam Okware supervised Uganda ministry of health staff, reviewed the protocol and submitted it for ethical approval and contributed in this article. All authors have read and approved the final manuscript.
